# Mercury in Orange Birch Bolete *Leccinum versipelle* and soil substratum: bioconcentration by mushroom and probable dietary intake by consumers

**DOI:** 10.1007/s11356-015-5331-8

**Published:** 2015-09-08

**Authors:** Grażyna Krasińska, Jerzy Falandysz

**Affiliations:** Laboratory of Environmental Chemistry & Ecotoxicology, Gdańsk University, 63 Wita Stwosza Street, 80-308 Gdańsk, Poland

**Keywords:** Foraging, Forest, Fungi, Mercury, Organic food, Soil

## Abstract

The aim of this study was to examine the contamination, accumulation, and distribution of mercury in fruiting bodies by *Leccinum versipelle* fungus collected from distant sites across Poland. Mercury was determined using validated method by cold-vapor atomic absorption spectroscopy after direct sample matrix combustion. A large set of data gained using 371 fruiting bodies and 204 soil samples revealed the susceptibility of *L. versipelle* to Hg contamination and permitted the estimation of probable intake of Hg contaminant by consumers foraging for this species. The range of median values of Hg determined in caps of *L. versipelle* was from 0.20 to 2.0 mg kg^−1^ dry biomass, and the median for 19 localities was 0.65 mg kg^−1^ dry biomass. The values of the Hg bioconcentration factor (BCF) determined for *L. versipelle* correlated negatively with Hg contents. Mercury in topsoil beneath *L. versipelle* ranged from 0.019 to 0.041 mg kg^−1^ dry matter for less-contaminated locations (BCF of 17 to 65 for caps) and from 0.076 to 0.39 mg kg^−1^ dry matter for more contaminated locations (BCF of 1.9 to 22). Fruiting bodies of *L. versipelle* collected in some regions of Poland if consumed in amount of 300 g in one meal in a week could provide Hg doses above the provisionally tolerable weekly intake (PTWI) value of 0.004 mg Hg kg^−1^ body mass, while regular consumptions for most of the locations were below the limit even with more frequent consumption. Also summarized are available data on Hg for three species of fungi of genus *Leccinum* foraged in Europe.

## Introduction

The Orange Birch Bolete or red-capped scaber stalk *Leccinum versipelle* (Fr. & Hök) Snell is one of macromycetes (mushrooms) in the Kingdom of Fungi that are popular in the Central and Eastern Europe and in the Scandinavia. Mushroom *L. versipelle* is also commonly known as *Leccinum testaceoscabrum* (Secr.) Sing. and is the most popular among the genus *Leccinum* collected in Poland (Gumińska and Wojewoda 1985). The fruiting body of the fungus, *L. versipelle*, is generally considered as good while its flesh quickly gets dark discoloration when it is hurt (cut), cooked (boiled/blanched and fried/roasted), or dried.

Another *Leccinum* species that is also common and annually foraged in Central and Eastern Europe is the Brown Birch Scaber Stalk *Leccinum scabrum*, but this is less valued and is usually dried before use because its stipe is somewhat tough and the mature fruiting body is smaller in size when compared to *L versipelle* (Falandysz and Bielawski 2007; Falandysz et al. 2007c).

Fungi are numerous in species and habitats, and this makes it challenging to build up a reasonable and credible database on their chemical composition (Falandysz and Borovička 2013). Macromycetes pick up metallic elements and metalloids from soil and other substrates in which mycelia live. Some of them can efficiently accumulate, and some can also hyperaccumulate certain metallic elements, metalloids, or nonmetals in fruiting bodies, e.g., Ag, As, V, and Se, when they emerge at background areas (Borovička et al. 2007; Falandysz et al. 2007a; Stijve et al. 1990 and Stijve et al. 1998). The fruiting bodies of certain species can be also rich in mercury (Hg) absorbed even from soil substrate whiteout Hg geochemical anomalies or anthropogenic pollution, and an example could be *Macrolepiota procera* or *Boletus edulis*, and some other *Boletus* mushrooms (Falandysz 2014; Falandysz et al. 2001b, 2003c, 2003d, 2007b, 2011, 2014b; Gucia et al. 2012; Melgar et al. 2009; Nasr et al. 2012; Ostos et al. 2015). Nevertheless, macromycetes differ in sequestration rate of Hg in fruiting bodies, and this could be considered as species-specific feature regardless of concentration in soil (Chudzyñski et al. 2009; Falandysz et al. 2014b and Falandysz et al. 2015c; Kojta et al. 2015; Krasińska and Falandysz 2015; Melgar et al. 2009; Nasr and Arp 2011).

The anthropogenic pollution of soils or other substrate with heavy metals (Ag, Hg), metalloids (As), or radionuclides (^137^Cs and others) usually causes their elevated accumulation in fruiting bodies of fungi (Falandysz et al. 1994a, 1994b, 2014a, 2015a and Falandysz et al. 2015b; García et al. 2015; Larsen et al. 1998). Fungi that grow on metallic/metalliferous soils or mine tailings can be specifically enriched in some elements (Ag, As, Cd, Hg, Pb) accumulated in fruiting bodies (Mleczek et al. 2015; Kojta et al. 2012 and Kojta et al. 2015; Niedzielski et al. 2013; Řanda and Kučera 2004). Those from soils contaminated because of Hg mining can sequester Hg in the emerged fruiting bodies at level as high as 471 mg kg^−1^ dry matter (dm) as observed in *Lactarius quietus* (Árvay et al. 2014).

The place in soil substratum where the mycelium lives seems to be key in understanding the overriding sources of Hg to fungus—a mineral horizon or organic horizon with decaying layer at the surface and humus below. This could be disturbed by metallic element depositions from atmospheric fallout especially where the soil structure favors quicker vertical passage of the element small molecules under consideration into the deeper layers of the soil alongside with infiltrating rain, which now takes a portion of elements/minerals that were not readily adsorbed by litter and organic horizon of soil into deeper layers. Certain fungi form mycelium and rhizomorphs (root-like structures), which seem to co-participate in the uptake and redistribution of Hg into fruiting bodies (Falandysz et al. 2013). Fungi having shallow mycelia can be efficient in the accumulation of airborne Hg deposited on the litter (Falandysz et al. 2014a).

Published data on annual rates of consumption of fungi collected from the wild by individuals are up to 20 kg per capita in the Great Britain and up to 20–24 kg per capita in Yunnan in China (Barnett et al. 1999; Zhang et al. 2010). Hence, the intake of minerals and other compounds contained in the flesh of fungi is of concern while our knowledge of their significance as sources of beneficial and noxious minerals is incomplete and worth of further study because of the high biodiversity of edible species and their wide distribution worldwide (Falandysz and Borovička 2013).

Mercury is a toxic chemical element, and because of the anthropogenic emissions of Hg into the atmosphere, it has recently become of greatest concern as a global contaminant of food and environment (Barnett et al. 1999; Blackwell and Driscoll 2015 ; Bushey et al. 2008; UNEP 2013). Many wild-grown fungi both of mycorrhizal and saprophytic nature can efficiently bioconcentrate Hg contained in substratum on which they emerge in their fruiting bodies (Drewnowska et al. 2012, 2014; Drewnowska and Falandysz 2015, 2014; Falandysz and Bielawski 2001; Falandysz and Brzostowski 2007; Fischer et al. 1995; Kojta et al. 2012; Rieder et al. 2011). The Hg contents in fruiting bodies of edible fungi are often elevated and much higher when compared to plants, while quantitative relationship on the redistribution of Hg between the mycorrhizal fungus and symbiont plant is not yet well known (Moore et al. 1995).

Foraging for edible and medicinal fruiting bodies of wild-grown fungi is a kind of gourmet tradition among various nations, while rates of consumption vary between the nations, populations, citizens and villages, families, and individuals (Barnett et al. 1999; Li et al. 2013; Nnorom et al. 2012; Petkovšek and Pokorny 2013; Tel et al. 2014). The *L. versipelle* is a mycorrhizal fungus, and the mycorrhizal fungi are known for their role in the redistribution of inorganic nutrients and other minerals to plants while many edible species are traditionally foraged from the wild in different regions of the world. This study aimed at elucidating the bioconcentration potential, distribution, and probable dietary intake and potential health risk for the local populations from Hg accumulated in fruiting bodies by *L. versipelle*, which emerged at 19 spatially and distantly distributed locations considered background areas (unpolluted forests) in Poland and which receive airborne Hg from global fallout that is dominated by anthropogenic emissions. The locations of material collection were without Hg parent soil bedrock anomaly. Also presented and discussed is a review of available data on Hg in this mushroom.

## Materials and methods

**Fig. 1:**
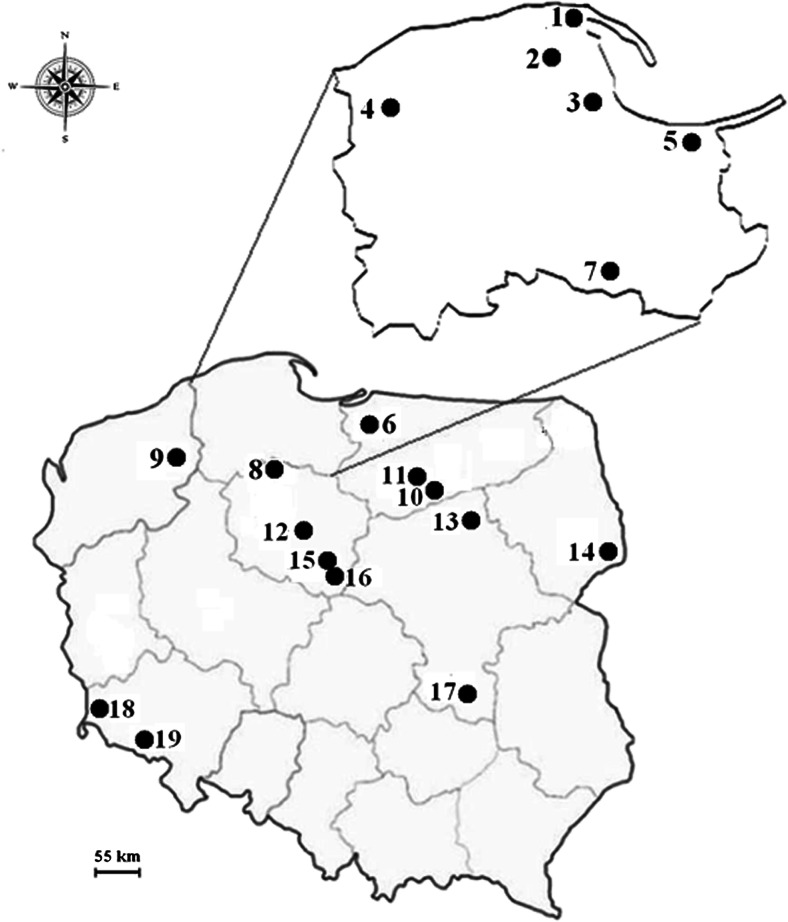
Localization of the sampling sites (1–19) of *L. versipelle* (for details, see in Table 1)

Specimens of the *L. versipelle* (Fr. & Hök) Snell fruiting bodies (371 individual samples) and 204 pooled samples of the upper 0–10-cm layer of forest soil (ca. 100 g) beneath the specimens were collected from 19 spatially distant localities in Poland in the period 1998–2008 (Fig. 1; sampling details shown in Table 1). Any plant materials and particles of soil adhered to the mushroom were removed from the surface of the fruiting bodies, while bottom part of the stipe was cut off using a plastic knife. Thereafter, the fruiting bodies were subsequently air-dried at room temperature in a well-ventilated room for 2–3 days. Thereafter, they were dried in an electrically heated oven at 65 °C until constant weight under clean laboratory condition. Dried fruiting bodies (separately caps and stipes) were then crushed and ground to a fine powder in a porcelain mortar. The soil substrate samples were air-dried at room temperature for approximately 10 weeks and then sieved through 2-mm sieve.

**Table 1:** Total mercury content of *Leccinum versipelle* and beneath soil substratum and values of mercury cap to stipe concentration quotient (*Q*_C/S_) and bioconcentration factor (BCF) (mg kg^−1^ dried fungal or soil material; arithmetic mean ± SD, median value and range, respectively)

Localization, year, and number of specimens	Hg (mg kg^−1^)			*Q*_C/S_	BCF	
	Caps	Stipes	Soils		Caps	Stipes
(1)^b^ Pomerania land, Nearshore Landscape Park, 2009, *n =* 19	0.49 ± 0.21 0.46 0.19–0.91	0.34 ± 0.19 0.29 0.18–0.90	0.019 ± 0.010 0.015 0.0094–0.049	1.5 ± 0.45 1.5 0.66–2.5	31 ± 18 26 5.5–83	21 ± 12 16 3.7–52
(1) Pomerania land, Nearshore Landscape Park, 2011, *n =* 16	0.57 ± 0.38 0.38 0.19–1.39	0.46 ± 0.20 0.39 0.15–0.86	WD	1.2 ± 0.5 1.0 0.7–2.3	WD	WD
(2) Pomerania land, Puszcza Darżlubska, 2003, *n =* 15	0.63 ± 0.30 0.59 0.25–1.5	0.41 ± 0.17 0.35 0.15–0.72	0.029 ± 0.010 0.033 0.012–0.038	1.6 ± 0.5 1.5 1.0–2.6	24 ± 13 20 8–60	16 ± 11 14 6–52
(3) Pomerania land, Trójmiejski Landscape Park, Gdańsk-Osowa 2006, *n =* 10 (13)^a^	0.91 ± 0.26 0.95 0.36–1.3	0.72 ± 0.21 0.79 0.25–0.89	0.027 ± 0.006 0.026 0.019–0.041	1.3 ± 0.2 1.3 1.1–1.6	35 ± 11 34 14–50	28 ± 11 29 9.5–47
(4) Pomerania land, Włynkowo, Dębica Kaszubska, 2003, *n* = 14 (46)	0.96 ± 0.35 0.95 0.39–1.7	0.79 ± 0.34 0.73 0.40–1.6	0.037 ± 0.020 0.031 0.020–0.078	1.3 ± 0.4 1.2 0.50–2.1	27 ± 15 20 13–58	23 ± 13 20 9.0–54
(5) Pomerania land, Stegna, 2006, *n =* 13	0.77 ± 0.30 0.75 0.42–1.3	0.44 ± 0.16 0.41 0.23–0.74	0.025 ± 0.002 0.025 0.021–0.029	1.8 ± 0.5 1.7 1.2–2.8	29 ± 12 27 14–50	16 ± 7 15 7.8–30
(6) Pomerania land, Elbląg, Młynary, 2006, *n =* 15 (26)	0.51 ± 0.14 0.53 0.21–0.70	0.40 ± 0.10 0.40 0.19–0.50	0.18 ± 0.05 0.19 0.045–0.22	1.3 ± 0.2 1.2 1.1–1.6	3.4 ± 2.6 2.9 1.0–12	2.7 ± 2.3 2.1 0.90–11
(7) Pomerania land, Bory Tucholskie, 2009, *n* = 15	0.60 ± 0.26 0.59 0.12–1.2	0.40 ± 0.20 0.36 0.19–1.0	WD	1.5 ± 0.5 1.7 0.41–2.4	WD	WD
(8) Pomerania land, Bory Tucholskie, Osiek, 2000, *n =* 19 (26)	0.64 ± 0.3 0.53 0.26–1.2	0.43 ± 0.15 0.40 0.24–0.80	WD	1.5 ± 0.4 1.5 0.66–2.1	WD	WD
(9) Pomerania land, Szczecinek, 2003, *n =* 9	0.63 ± 0.12 0.63 0.41–0.79	0.46 ± 0.08 0.45 0.32–0.57	WD	1.4 ± 0.3 1.5 1.0–1.7	WD	WD
(10) Warmia land, Puchałowo 2001, *n* = 1 (14)	1.0	0.86	WD	1.2	WD	WD
(10) Warmia land, Puchałowo 2004, *n* = 1 (8)	0.79	0.68	WD	1.2	WD	WD
(11) Warmia land, Puszcza Napiwodzko-Ramucka, 2003, *n =* 15	1.3 ± 0.5 1.2 0.64–2.2	0.72 ± 0.21 0.79 0.24–0.89	0.027 ± 0.006 0.026 0.019–0.041	1.6 ± 0.3 1.6 1.1–2.3	65 ± 37 65 7.9–160	42 ± 26 40 5.1–93
(12) Kujawy land, Toruń outskrits, 2000, *n =* 16	0.35 ± 0.15 0.31 0.20–0.79	0.17 ± 0.07 0.15 0.080–0.34	0.044 ± 0.029 0.041 0.0079–0.17	1.8 ± 0.5 1.7 1.4–2.6	8.6 ± 5.7 8.6 3.1–14	4.8 ± 3.4 3.4 1.9–9.3
(13) Mazowsze land, Commune Olszewo-Borki, 2007, *n =* 12	0.36 ± 0.46 0.36 0.15–1.4	0.32 ± 0.25 0.24 0.097–0.87	0.024 ± 0.014 0.019 0.0093–0.054	1.5 ± 0.3 1.5 1.1–2.1	25 ± 22 18 3.5–74	18 ± 15 12 2.2–45
(14) Podlasie land, Puszcza Białowieska, Białowieża, 1998, *n =* 10	0.29 ± 0.12 0.26 0.14–0.46	0.17 ± 0.07 0.15 0.089–0.30	0.089 ± 0.049 0.082 0.031–0.18	1.7 ± 0.37 1.7 1.3–2.6	4.6 ± 4.2 3.0 0.79–15	2.7 ± 2.7 1.6 0.60–9.0
(15) Kujawy land, Włocławek outskrits, 2004, *n =* 13	0.70 ± 0.30 0.66 0.31–1.3	0.43 ± 0.12 0.42 0.22–0.66	0.019 ± 0.003 0.018 0.013–0.024	1.6 ± 0.3 1.6 1.0–2.2	37 ± 15 30 17–75	23 ± 6 22 16–39
(16) Kujawy land, Gostynińsko-Włocławskie forests, Goreń, 2001, *n =* 12 (27)	0.58 ± 0.26 0.59 0.17–0.97	0.41 ± 0.20 0.35 0.13–0.83	0.030 ± 0.023 0.023 0.014–0.099	1.4 ± 0.4 1.3 1.2–2.6	16 ± 7 17 3.6–27	24 ± 14 23 8.6–48
(17) Świętokrzyskie land, Starachowickie forests, Bugaj, 2000, *n =* 15 (48)	2.0 ± 0.4 2.0 1.2–2.5	1.1 ± 0.3 1.1 0.60–1.7	0.098 ± 0.038 0.076 0.055–0.16	1.8 ± 0.3 1.8 1.3–2.4	23 ± 10 22 10–39	13 ± 7 10 5–26
(18) Zgorzelec, 2004, *n =* 1 (7)	1.3	0.75	WD	1.7	WD	WD
(19) Lower Silesia land, Karkonosze Mts., Karpacz, 2008, *n =* 3	0.97 ± 0.64 0.74 0.48–1.7	0.74 ± 0.40 0.55 0.46–1.2	0.38 ± 0.01 0.39 0.37–0.39	1.3 ± 0.2 1.3 1.0–1.4	2.6 ± 1.8 1.9 1.2–4.6	1.9 ± 1.1 1.4 1.2–3.1

*WD* without data

^a^Number of pooled samples and number of specimens (in parentheses)

^b^Localization shown in Fig. 1

Total Hg content of the materials was determined after a direct sample thermal decomposition and released Hg vapor concentration measurement by cold-vapor atomic absorption spectroscopy (CV-AAS) (Mercury analyzer type MA-2, Nippon Instruments Corporation, Takatsuki, Japan). Analytical control and quality assurance (AC/QA) were performed by the analysis of certified reference material CS-M-1 (dried fruiting bodies of fungus Cow Bolete *Suillus bovinus*) and CS-M-4 (dried fruiting bodies of fungus Common Birch Stalk *L. scabrum*) produced by the Institute of Nuclear Chemistry and Technology in Warsaw, Poland. The Hg content of CS-M-1 is certified as 0.174 ± 0.018 mg kg^−1^ dm and of CS-M-4 is certified as 0.465 ± 0.024 Hg mg kg^−1^ dm, while our measurement during the analysis of *L. versipelle* and the substrates showed 0.179 ± 0.024 mg kg^−1^ dm (*n* = *23*) for CS-M-1 and 0.449 ± 0.026 mg kg^−1^ dm for (*n* = *11*) CS-M-4. For every set of ten fruiting bodies or soil samples analyzed, one blank sample was examined (Chudzyński et al. 2011; Jarzyńska and Falandysz 2011; Nnorom et al. 2013). The value of the quotient between Hg content of fruiting body and soils (the top layer in which mycelium develops and from which it takes up minerals) is considered as the bioconcentration factor (BCF) (Falandysz and Borovička 2013).

Table 1 summarizes information on Hg determinations (mg kg^−1^ dry matter (dm)) in *L. versipelle* and soil substratum as well as the calculated values of quotient of Hg in cap to stipe concentration (*Q*_C/S_) and the bioconcentration factor (BCF) from this study. Table 2 summarizes the available data on occurrence on Hg in morphological parts or whole fruiting bodies of three species of genus *Leccinum* (*Leccinum rufum*, *L. scabrum*, and *L. versipelle*) collected in Europe. The analytical results were statistically treated using computer software Statistica (version 8.0) by StatSoft. Because mercury concentration in fruiting bodies has no Gaussian distribution (Chudzyñski et al. 2009), the possible statistically significant differences between the variables were examined with the aid of the nonparametric Mann–Whitney *U* test.

**Table 2:** Mercury (mg kg^−1^ dry biomass) in certain mushrooms of genus *Leccinum* in Europe (adapted)

Species, year(s), part of fruiting body, and number of individuals	Mean/s ± SD	Median/s	Total range	Reference
*L. rufum* (caps), 1997/1998, *n =* 117	0.60 ± 0.59–1.8 ± 0.6	0.51–1.7	0.28–2.8	^abcde^
*L. rufum* (caps), 2004/2006, *n =* 126	0.27 ± 0.07–1.3 ± 0.6	0.28–1.3	0.16–2.2	^f^
*L. rufum* (stipes), 1997/1998, *n =* 117	0.45 ± 0.24–1.7 ± 0.6	0.42–1.6	0.067–2.7	^abcde^
*L. rufum* (stipes), 2004/2006, *n =* 126	0.19 ± 0.06–0.58 ± 0.08	0.18–0.54	0.077–0.89	^f^
*L. scabrum* (caps), 1994, *n* = 16	0.37 ± 0.33	0.27	0.093–1.2	^g^
*L. scabrum* (caps), 1994, *n* = 16	0.29 ± 0.30	0.20	0.12–1.3	^h^
*L. scabrum* (caps), 1993/1994, *n* = 14	0.29 ± 0.10	0.28	0.14–0.46	^i^
*L. scabrum* (caps), 1995, *n =* 19	0.18 ± 0.13	0.19	0.033–0.47	^j^
*L. scabrum* (caps), 1995, *n* = 15	0.50 ± 0.23	0.46	0.17–0.93	^k^
*L. scabrum* (caps), 1997/1998, *n =* 12	6.7 ± 2.2	7.3	3.2–9.6	^l^
*L. scabrum* (caps), 1998/2001, *n =* 240	0.38 ± 0.23–1.2 ± 0.4	0.36–1.2	0.072–2.0	^m^
*L. scabrum* (stipes), 1994, *n* = 16	0.22 ± 0.16	0.17	0.050–0.65	^g^
*L. scabrum* (caps), 1994, *n* = 16	0.18 ± 0.16	0.14	0.052–0.64	^h^
*L. scabrum* (stipes), 1993/1994, *n* = 14	0.18 ± 0.06	0.18	0.061–0.29	^i^
*L. scabrum* (stipes), 1995, *n =* 19	0.10 ± 0.06	0.11	0.015–0.21	^j^
*L. scabrum* (stipes), 1995, *n* = 15	0.32 ± 0.13	0.30	0.14–0.64	^k^
*L. scabrum* (stipes), 1997/1998, *n =* 12	4.6 ± 1.7	4.6	1.7–7.7	^l^
*L. scabrum* (stipes), 1998/2001, *n =* 240	0.17 ± 0.07–1.1 ± 0.3	0.080–1.1	0.034–1.7	^m^
*L. scabrum*(h/rfb), 2005/2006, *n =* 6	0.57 ± 0.23 / 0.44 ± 0.31	–	–	^n^
*L. scabrum* (whole fruit bodies) *n =* 15	0.33 ± 0.21	0.24	0.13–0.93	^d^
*L. versipelle* (whole), p. 1976	0.37	–	0.27–0.45	^o^
*L. versipelle* (caps), 1995/1997, *n =* 15	0.46 ± 0.04	0.35	0.080–1.7	^a^
*L. versipelle* (caps), 1995, *n =* 13	0.50 ± 0.31	0.51	0.010–1.2	^i^
*L. versipelle* (caps), 1997/1998, *n* = 16	0.72 ± 0.38	0.67	0.28–1.7	^p^
*L. versipelle* (caps), 1998/2008, *n =* 257	0.20 ± 0.07–2.0 ± 0.4	0.20–2.0	0.14–2.5	This study
*L. versipelle* (stipes), 1995/1997, *n =* 31	0.25 ± 0.22	0.14	0.052–0.91	^a^
*L. versipelle* (stipes), 1995, *n =* 13	0.30 ± 0.23	0.30	0.057–0.94	^i^
*L. versipelle* (caps), 1997/1998, *n* = 16	0.42 ± 0.38	0.43	0.16–0.89	^p^
*L. versipelle* (stipes), 1998/2008, *n =* 257	0.17 ± 0.07–1.1 ± 0.3	0.080–1.1	0.034–1.7	This study
*Leccinum* sp*.* (whole), p. 1998	0.26		^q^	

*h/rfb* hymenophore/rest of fruit body, *p*. year of publication

^a^Falandysz (2002)

^b^Falandysz et al. (2002b)

^c^Falandysz et al. (2003a)

^d^Falandysz et al. (2002c)

^e^Falandysz et al. (2004)

^f^Falandysz et al. (2012b)

^g^Falandysz et al. (1996)

^h^Falandysz and Kryszewski (1996)

^i^Falandysz and Chwir (1997)

^j^Falandysz et al. (2001a)

^k^Falandysz et al. (2003c)

^l^Falandysz et al. 2003b

^m^Falandysz and Bielawski 2007

^n^Melgar et al. (2009)

^o^Seeger and Nützel (1976)

^p^Falandysz et al. (2002a)

^q^Tüzen et al. (1998)

## Results and discussion

### Contents and efficiency of Hg sequestering in *L. versipelle* fruiting body

The values of Hg BCF for all samples (specimens) in this study were above 1, and for some of the sites, the median values for caps were up to 65 (Table 1). This implies that there is a good bioconcentration potential of Hg by *L. versipelle*. The absorption of Hg by this fungus from sources other than soils, i.e., air, due to airborne deposition of Hg onto the surface of fruiting bodies can be neglected. This is because the concentration of Hg in atmospheric air over the background (uncontaminated) areas and regions of the World is low, e.g., in Europe is from 2 to 3 ng per cubic meter in summer and from 3 to 4 ng per cubic meter in winter, and for the urbanized areas, this is about 3–4-fold higher (WHO 2000). The fruiting bodies of macromycetes emerge quickly and, depending on species, become matured in one or more days, and their lifetime is relatively short.

In aquatic environment, methylmercury in a complex with amino acid cysteine is well accumulated and biomagnified by biota in a food chain (UNEP 2013). This remains unknown for fungi. Both inorganic Hg and MeHg are efficiently accumulated by numerous species of mushrooms (Stijve and Roschnik 1974). An inorganic Hg is the dominant form in the total Hg sequestered in fruiting bodies while MeHg is minor. For example, in *Leccinum griseum*, the MeHg accounted at 0.5 % in total Hg (Rieder et al. 2011), while no such data are available for *L. versipelle*.

The reported values of the Hg BCF determined for caps and stipes of *L. versipelle* correlated negatively with the median values of Hg content in the top fraction of soils which, for four sites, ranged from 0.076 to 0.39 mg kg^−1^ dm (BCF of 1.9–22 for caps) and, for 14 other sites, ranged from 0.019 to 0.041 mg kg^−1^ dm (BCF of 17–65 for caps) (Table 1).

Earlier, it has been found that efficiency of Hg inclusion into fruiting bodies by certain fungi that emerged at an “unpolluted” soil occurred with considerably greater efficiency compared to those that emerged at “polluted” areas (Falandysz et al. 2012a, 2012b, 2012c, 2012d). This could be observed also for *L. versipelle* from Puszcza Napiwodzko-Ramucka and the Starachowicki forests in this study, which contained Hg, respectively, in caps at 1.3 ± 0.5 and 2.0 ± 0.4 mg kg^−1^ dm and in stipes at 0.72 ± 0.21 mg kg^−1^ dm, while efficiency of inclusion measured by value of BCF was 65 ± 37 and 23 ± 10 (caps) and 42 ± 26 and 13 ± 7 (stipes), and soil Hg was at 0.027 ± 0.006 and 0.098 ± 0.038 mg kg^−1^ dm.

There was a clear difference (0.001 < *p* < 0.05; Mann–Whitney *U* test) in Hg contamination of both the caps and stipes of *L. versipelle* for some of the sites studied, and range for caps was from 0.29 ± 0.12 mg kg^−1^ dm (Puszcza Białowieska) to 2.0 ± 0.4 mg kg^−1^ dm (Starachowickie forests) and for stipes from 0.17 ± 0.07 to 1.1 ± 0.3 mg kg^−1^ dm, while for majority of the specimens, Hg content for caps was around 0.6 mg kg^−1^ dm and that of stipes about 0.4 mg kg^−1^ dm (Table 1).

The *L. versipelle* (Tables 1 and 2) and many other species of wild-grown fungi are substantially more enriched with Hg compared to the edible plants (leafy vegetables, fruits, tubers, and grains) or tissues and organs of slaughtered animals as well as in certain sea foods (Falandysz 1994; Falandysz et al. 1994c; Guédron et al. 2014; Varo et al. 1980). Hence, fruiting bodies of *L. versipelle* can be of a concern as a source of exposure to Hg by consumers.

### Hg in some *Leccinum* spp. and Hg intake

Generally, the fruiting bodies (and also the sclerotia) of edible fungi collected from the wild are considered as delicacy and even as having a medicinal value in various parts of the World (Dai et al. 2013; Nnorom et al. 2013). The species *L. versipelle* as well as *L. rufum* and young *L. scabrum* are tasty, popular, and valuable wild-grown fungi used traditionally as gourmet in Central and Eastern Europe and in Scandinavia. The Yunnan land of China has a wide variety of species and dishes of fungi (Wang et al. 2014). Similarly, within the “Western” societies, the wild-grown fungi are also components of rather exclusive meals (Lassoe et al. 1996).

Amongst of the *Leccinum* mushrooms that are edible and popular in the Central and Eastern Europe and Scandinavia, apart from the *L. versipelle*, are species such as *L. scabrum* (Bull.: Fr.) S.F. Gray and *L. rufum* (Schaeff.) Kreisel, which was also called *Leccinum aurantiacum* (Bull.) Gilb., and less or much less frequent are *Leccinum pseudoscabrum* (Kallenb.) Šutara [earlier known as *L. griseum* (Quél.) Singer 1966 and *Leccinum carpini* (R. Schulz) M.M. Moser ex D.A. Reid 1965], *Leccinum variicolor* Watling 1969, *Leccinum vulpinum* Watl., *Leccinum piceinum* Pilát & Derm., *Leccinum quercinum* (Pilát) Green et al. Watl*.*, and *Leccinum duriusculum* (Kalchbr.) (Singer, 1947) (Gumińska and Wojewoda 1985; Jarzyńska and Falandysz 2012a, 2012b). Data available on Hg content and distribution in fruiting bodies of *L. rufum*, *L. scabrum*, and *L. versipelle* collected in Europe and including unidentified *Leccinum* sp. from Turkey are summarized in Table 2.

As can be noted from Table 2, in several studies with different number of specimens and localities examined, the species *L. rufum* with the Hg median values in caps from 0.18 to 1.7 mg kg^−1^ dm and *L. scabrum* with from 0.11 to 1.2 mg kg^−1^ dm, all showed roughly similar contents of Hg as found in *L. versipelle* in this study*.* One exception from that pattern seems to be a median value of 7.3 mg kg^−1^ dm reported for caps of *L. scabrum* from one site that was considered as being under anthropogenic impacts and most probably related to the World War II and/or other military activities in the past (Table 2). Hence, in general, an intake and risk from Hg contained in flesh of these species mirror comments drawn above for *L. versipelle*.

Loss of Hg from the flesh of fungi when cooked is relatively low (around 10 %) if an ordinary processing procedure (e.g., blanching for 10 min) is applied (Falandysz and Drewnowska 2015), and this needs further studies because of several procedures known (freezing, frying, grilling, pickling, salting/souring) with possible impact on Hg and other trace elements and minerals content of cooked fruiting bodies.

### *L. versipelle* and Hg intake

For the assessment of the risks due to intake of inorganic Hg from food, one of the parameters used is the Hg reference dose (RfD) of 0.0003 mg kg^−1^ body mass (bm) daily (US EPA 1987). For this purpose, the provisional tolerable weekly intake (PTWI) of THg is also used (i.e., 0.004 mg kg^−1^ bm) (JECFA 2010).

Because of the important biological and geochemical interactions of Hg and selenium (Se), it was suggested recently that the assessment of risk from Hg should consider the interaction between Hg and Se (Ralston and Raymond 2014; Zhang et al. 2014). Selenium is at ultra-trace levels in food and is essential at very small amounts, while there is a high risk of toxicity if taken in excess. Selenium is an antagonist to MeHg and inorganic Hg, which is toxic, but a common contaminant of foods which at ultra-trace level is considered without biological effect. A possible protective role of Se against the biological activity of Hg contained in mushrooms and vice versa is worth of future studies.

In some nations, the edible sclerotia of some fungi are also popular (Nnorom et al. 2013; Wang et al. 2015). Apart from the different consumption patterns of fungi between individuals and populations worldwide, another factor involving variability is the earlier mentioned biodiversity of species with many having, to some extent, a cosmopolitan character while others are having more local or regional distribution. Another variable is the distinct differences in natural contents of particular elements in mature fruiting bodies of a given species of fungus that emerged at spatially distant sites as shown for Hg and *L. versipelle* in this study (Table 1). Data concerning spatial differences and similarities in fruiting body contents of Hg or other trace elements and minerals are rarely published (Brzostowski et al. 2011a and Brzostowski et al. 2011b; Chojnacka et al. 2012, 2013; Chudzyński and Falandysz 2008; Falandysz et al. 2007a, 2007b, 2007d).

It is reasonable to assume that an average amount of fresh fruiting bodies consumed in a meal by a fancier is 300 g while some could consume as much as 500 g (Chudzyński et al. 2011). For some individuals who may be characterized by the maximum annual consumption of wild-grown fungi (estimated at 20–24 kg as reported until now), the annual intake would be from around 67 to 80 meals each of 300-g fruiting bodies.

In this study, the range of median values of Hg determined in caps of *L. versipelle* per site was from 0.20 to 2.0 mg kg^−1^ dm and the median value for all 19 localities was 0.65 mg kg^−1^ dm (Table 1). Assuming that moisture content of the fresh fruiting body is 90 %, the contents of Hg for fresh caps are 0.02, 0.20, and 0.065 mg kg^−1^, respectively, and around 1.5-fold less in the stipes.

A meal made of 300 g of fresh caps of *L. versipelle* from the areas examined could provide from 0.006 to 0.060 mg Hg (a dose of 0.0001 to 0.0010 mg/kg bm for person of 60 kg bm), and the median Hg intake for all 19 locations studied would be 0.0195 mg (0.000325 mg kg^−1^ bm) assuming no Hg intake from other sources. Theoretically, if eaten daily, the Hg intake rate calculated for the “average” and “maximum” contaminated mushrooms is equal to or exceeds the RfD value by 3-fold. Nevertheless, fruiting bodies of wild-grown fungi are not eaten daily even by fanciers or villagers in Poland but certainly can be eaten by them more frequently in mushrooming season that is from July to October.

In light of the value of PTWI for inorganic Hg, one meal consisting of caps of *L. versipelle* for the sites contaminated at the average or maximum level as shown in this study will provide Hg at 20 to 60 % of the PTWI. Hence, depending on the area of fungi pickup, more than one meal in a week could result in exceeding the PTWI. A less-frequent consumption could be considered safe.

## Conclusions

The Orange Birch Bolete (*L. versipelle*) has good potential to accumulate Hg from soils. Specimens of this *L. versipelle* collected at background (unpolluted) areas in Poland showed Hg at levels that could be considered as safe and probably representative for species in this part of Europe.
